# ACL injury in female soccer players: Risk, resilience, and prevention in the modern game

**DOI:** 10.1177/00368504261428994

**Published:** 2026-04-09

**Authors:** Riccardo D’Ambrosi, Philipp Baumert, Christian Fink

**Affiliations:** 1 46767 IRCCS Ospedale Galeazzi—Sant’Ambrogio, Milan, Italy; 2 207131 Link Campus University, Rome, Italy; 3 Research Unit for Orthopaedic Sports Medicine and Injury Prevention, 31510 Institute for Sports Medicine, Alpine Medicine and Health Tourism, Private University for Health Sciences, Hall in Tirol, Austria; 4 School of Sport and Exercise Sciences, Liverpool John Moores University, Liverpool, UK; 5 722271 Gelenkpunkt-Sports and Joint Surgery FIFA Medical Centre of Excellence, Innsbruck, Austria; 6 Research Unit for Orthopaedic Sports Medicine and Injury Prevention (OSMI), Private University for Health Sciences Medical Informatics and Technology, Innsbruck, Austria

**Keywords:** Anterior cruciate ligament injury, female soccer players, neuromuscular control, return to sport, injury prevention, biomechanics, graft choice, osteoarthritis risk

## Abstract

Anterior cruciate ligament (ACL) injury is one of the most impactful conditions in female soccer, with major consequences for knee function, osteoarthritis risk, and professional longevity. As participation and competitive demands in women's football continue to rise, the disproportionate ACL burden has become a critical clinical and public health concern.

This narrative review provides a contemporary synthesis of current evidence on ACL injury in female soccer players, integrating data on epidemiology, injury mechanisms, intrinsic and extrinsic risk factors, surgical outcomes, return to sport (RTS), and prevention strategies. Across multiple cohorts, female players sustain ACL injuries at rates two to nine times higher than males, predominantly through noncontact mechanisms. Video analyses indicate that approximately 90% of injuries occur during cutting, pressing, deceleration, or landing tasks.

Key intrinsic risk factors include a reduced hamstring-to-quadriceps strength ratio, quadriceps dominance, generalized joint laxity, hip abductor weakness, and longer playing experience. Biomechanical deficits such as dynamic knee valgus, trunk instability, and suboptimal change of direction mechanics further increase susceptibility. Psychological factors, particularly fear of reinjury, also influence movement patterns and RTS.

Although ACL reconstruction generally yields favorable outcomes, reinjury remains a major concern in athletes returning to pivoting sports, with reported RTS rates ranging from 70% to 78%. Neuromuscular-based prevention programs, including FIFA 11+-derived protocols, can reduce ACL incidence by 40–45% when consistently implemented; however, adherence and limited sex-specific tailoring remain significant barriers.

ACL injury in female soccer players is a multifactorial and largely preventable condition, requiring integrated, sex-specific prevention and rehabilitation strategies to effectively reduce risk and support long-term athletic health.

## Introduction

Women's soccer has experienced rapid global expansion, with more than 30 million female participants and major professional leagues in Europe and North America reaching unprecedented competitive standards. Parallel to this growth, a concerning epidemiological trend persists: female soccer players sustain anterior cruciate ligament (ACL) injuries at approximately two to nine times the rate of their male counterparts, as demonstrated across recent large cohort studies in professional football.^1–3^

The ACL plays a central role in knee stability and proprioceptive function, and its rupture disrupts the kinetic chain required for high-speed cutting, deceleration, and landing tasks. Consequences extend well beyond time loss, often including prolonged physical deficits, performance decline, and an elevated risk of posttraumatic osteoarthritis (OA).^4,5^ For many elite female players, an ACL injury represents a defining moment in their career—several do not return to their preinjury level, and women are more likely than men to retire earlier following a major knee injury.^3,6^

This narrative review synthesizes current evidence on ACL injury in female soccer players, integrating epidemiology, mechanisms, intrinsic and extrinsic risk factors, surgical outcomes, rehabilitation, and prevention strategies. The goal is to provide a clinically relevant, evidence-informed resource for orthopedic surgeons, sports medicine physicians, physiotherapists, and coaches involved in the care and performance management of female athletes.

### Methods

This review is guided by the Scale for the Assessment of narrative review articles.^7^

The Transparency In The reporting of Artificial Intelligence checklist was completed to ensure transparent reporting of artificial intelligence use.^8^

This narrative review was conducted using a structured literature search strategy aimed at identifying contemporary evidence on ACL injury in female soccer players.

The databases PubMed/MEDLINE, Scopus, and Web of Science were searched from January 2000 to October 2025. The following key terms and their combinations were used: “*ACL injury*,” “*female soccer*,” “*women football*,” “*anterior cruciate ligament reconstruction*,” “*return to sport*,” “*risk factors*,” “*prevention*,” “*biomechanics*,” “*hormones*,” *and* “*osteoarthritis*.”

Only studies published in English were considered. Eligible article types included prospective and retrospective cohort studies, case-control studies, randomized and nonrandomized clinical trials, systematic reviews, and narrative reviews when relevant to underrepresented topics. Editorials, surgical technique articles, conference abstracts, dissertations, and non–peer-reviewed sources were excluded.

Studies were selected based on relevance to female soccer players and to at least one of the following domains: incidence, injury mechanisms, risk factors, hormonal influences, imaging, graft choice and lateral extra-articular procedures (LEAPs), return to play, psychological aspects, professionalization mismatch, long-term outcomes, and prevention.

To enhance transparency, a descriptive overview of the literature search and study selection process was performed. The initial search across PubMed/MEDLINE, Scopus, and Web of Science yielded a total of 76 records. After removal of duplicates (*n* = 20), titles and abstracts were screened for relevance to female soccer players and ACL-related outcomes.

**Figure 1.**
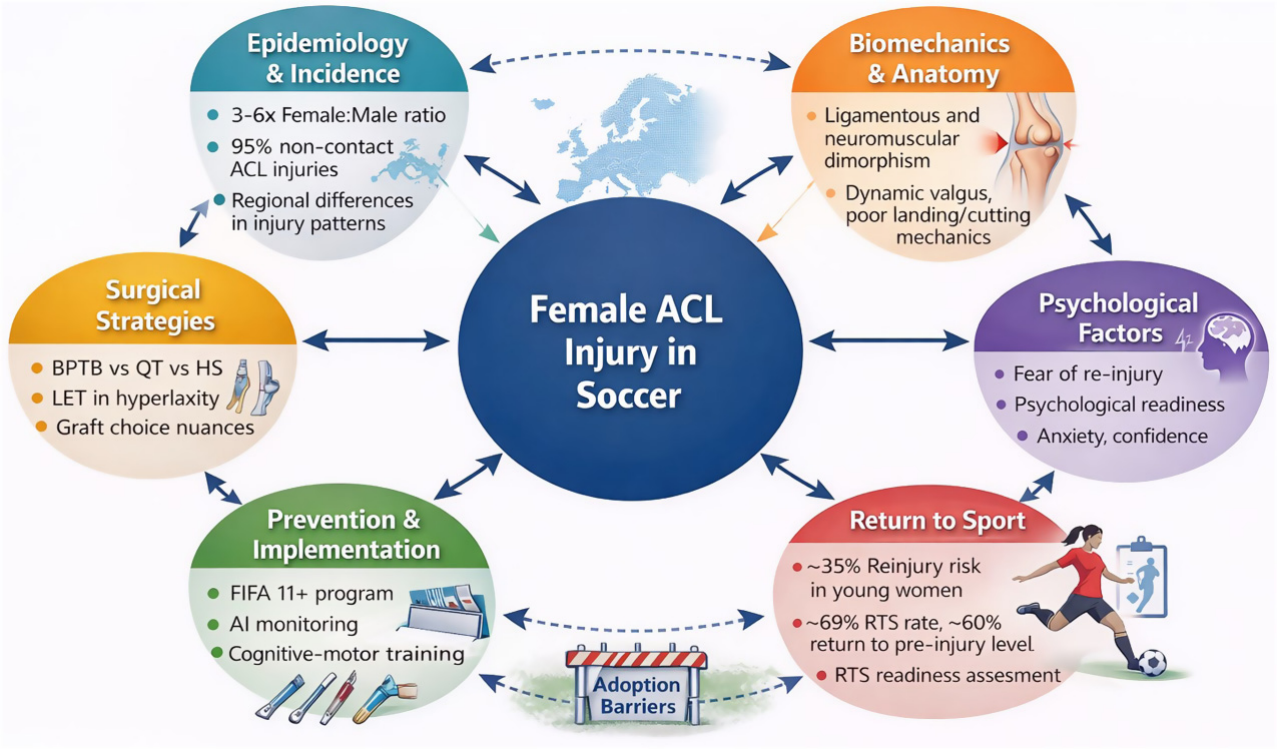
Conceptual framework of evidence synthesis across domains in female ACL injury research.

Articles were excluded at this stage primarily due to: (1) non–soccer-specific populations, (2) mixed-sex cohorts without sex-stratified data, (3) absence of ACL-specific outcomes, (4) nonoriginal study designs (editorials, conference abstracts), or (5) nonclinical or biomechanical relevance (Figure 1).

This figure illustrates the integrative framework used in this narrative review to synthesize evidence on anterior cruciate ligament (ACL) injury in female soccer players. ACL injury is depicted as the central outcome, informed by multiple interconnected domains, including epidemiology and incidence, biomechanics and anatomy, psychological factors, surgical strategies, return to sport, and prevention and implementation. The bidirectional relationships emphasize that individual studies may contribute to more than one domain, reflecting the multifactorial and interactive nature of ACL injury risk, management, and prevention in female soccer players.

Full-text articles were subsequently assessed for eligibility, with additional exclusions due to insufficient female-specific reporting, lack of sport-specific applicability, or redundancy with higher-quality or more recent evidence. Owing to the narrative design of this review, study selection was guided by relevance, methodological rigor, and clinical applicability rather than formal quantitative synthesis (Supplemental Table S1).

This review was intentionally designed as a narrative review rather than a systematic or scoping review, with the aim of offering an integrative, multidisciplinary, and clinically applicable synthesis of the available literature.

Owing to the narrative design of the review, a formal risk-of-bias assessment was not performed. All studies deemed relevant to female soccer players and ACL-related outcomes were considered. Greater emphasis was placed on prospective studies, larger cohorts, and sport-specific female populations when synthesizing and contextualizing the evidence, while studies with lower methodological rigor or limited applicability were included primarily for background or hypothesis-generating purposes rather than for drawing definitive conclusions. A descriptive overview of the study identification and selection process, including reasons for exclusion at each stage, is provided in Supplemental Table S1 to enhance transparency.

### Incidence

Incidence rates of ACL injury in female soccer players vary substantially across competition levels, but consistently indicate a higher burden compared with their male counterparts. In the Union of European Football Associations (UEFA) Women's Elite Club Injury Study, which prospectively followed 15 elite teams across four consecutive seasons, ACL injuries represented only 2% of all reported injuries but accounted for the highest injury burden, with a median of 292 days lost per injury (IQR 246–334).^9^

Although the study reported overall injury incidence (6.7 injuries per 1000 h), it could not provide sex-specific ACL incidence rates, as the cohort consisted exclusively of women's teams. Nevertheless, the high injury burden highlights the substantial impact of ACL rupture in elite female players.

Data from national top-level leagues reinforce this trend. In the Finnish Women's National League, a four-season prospective cohort reported 189 sudden-onset injuries, with knee injuries representing one of the most common categories and ACL injuries contributing substantially to total time-loss (mean 260.0 [IQR 170.0–354.0] days lost per ACL injury).^10^

A broader epidemiological perspective is provided by the six-season longitudinal registry from Catalonia, which included over 780,000 player-seasons across all competitive levels. ACL injury incidence was 0.43%, with a marked sex difference: female players demonstrated an incidence of 1.06% compared with 0.38% in males (Relative Risk 2.79).^11^

Notably, sex disparities emerged only after age 14, suggesting a combined influence of biological maturation and sport-specific exposure patterns. This large-scale dataset also identified seasonal peaks in October and January, with similar temporal patterns between sexes. This sex-specific divergence may be partly explained by maturational changes. A large longitudinal study demonstrated that knee abduction moments increase in females during and after peak height velocity, while remaining stable or decreasing in males, providing a biomechanical basis for the increased ACL injury risk observed in postpubertal female athletes.^12^

Finally, adolescent data from a systematic review and meta-analysis confirm that female athletes—particularly soccer players—exhibit the highest ACL incidence among youth sports. Across 19.1 million athlete-exposures, adolescent females had a higher ACL incidence (Incidence Rate 0.089 per 1000 athlete-exposyres) than males (Incidence Rate 0.062), with the greatest relative risk observed in soccer (Risk Ratio 3.12).^13^

Per-season risk in female soccer players reached 1.08%, the highest among all female adolescent sports.^13^

Collectively, these findings demonstrate that female soccer players across elite, national, and adolescent levels consistently show elevated ACL injury incidence and burden, highlighting the unique vulnerability of this population and the need for targeted prevention strategies.

## Injury mechanisms

ACL injuries in female soccer players arise predominantly from noncontact and indirect-contact situations, characterized by rapid deceleration, cutting, and pressing actions that impose multiplanar loads on the knee. Systematic video analyses consistently identify horizontal movement patterns, such as sprinting, pressing, tackling, and reactive deceleration, as the primary situational triggers, rather than vertical landings.^14^

Recent evidence shows three recurrent noncontact/indirect patterns: pressing/tackling, being tackled, and dribbling, each contributing distinct loading profiles, with pressing/tackling accounting for ∼42% of mechanisms and dribbling emerging as a newly described high-risk scenario.^15^

Across studies, ACL ruptures typically occur with a biomechanical constellation involving limited knee flexion (<30° at initial contact), progressive valgus collapse, and increased internal rotation moments, often accompanied by hip abduction >20° and trunk lateral flexion toward the injured side, all of which amplify knee abduction loads.^15^

This multiplanar collapse pattern aligns with prospective biomechanical work showing that players who later sustain ACL injuries demonstrate greater dynamic valgus, reduced knee flexion, contralateral trunk tilt, pelvic drop, and internal foot rotation during change of direction tasks.^16^

Trunk and pelvic instability are repeatedly highlighted as critical amplifiers of distal joint loads, underscoring the importance of proximal control. Fatigue and neurocognitive demands further exacerbate these risky mechanics. Pivot-turn analyses show that unanticipated conditions increase hip flexion/abduction, knee internal rotation, and peak ground reaction forces, while fatigue amplifies these demands and compromises coordination, leading to higher ACL-relevant loading during the braking phase of pivot turns.^17^

Combined fatigue–anticipation scenarios produce the *worst-case biomechanical profile*, with reduced knee flexion, increased internal rotation, and diminished ankle stability.^17^

Similar findings appear in change of direction tasks with increasing cognitive decision complexity, where additional movement options alter proximal joint mechanics—greater hip internal rotation/abduction and altered trunk rotation—demonstrating that cognitive load disrupts whole-body control even without large changes in peak knee moments.^18^

Additional mechanistic insights come from controlled laboratory assessments. Vertical drop-jump analyses reveal inter-limb asymmetries in rotation and ankle motion, indicating that asymmetrical loading and knee valgus tendencies predispose players to noncontact rupture mechanisms.^19^

Change of direction EMG studies in futsal athletes show that players at risk exhibit altered tibial rotation velocities and increased vastus medialis and gastrocnemius activity, suggesting a dysfunctional motor strategy that may heighten ACL loading during the load phase of change of direction maneuvers.^20^

Pressing-oriented tactical analyses similarly confirm immediate re-aggression (i.e. counter-pressing or rapid defensive pressure following ball loss) and other high-pressure defensive actions.^15^

Taken together, the 11 included studies converge on a unified model: ACL injuries in female soccer result from a combination of reactive, cognitively demanding horizontal movements performed under suboptimal neuromuscular control, characterized by valgus-internal-rotation collapse, reduced flexion angles, proximal instability, and fatigue-driven degradation of mechanics. These findings emphasize the need for prevention strategies targeting: (1) anticipatory and reactive neuromuscular training, (2) trunk–pelvis control, (3) change of direction technique under cognitive load, and (4) fatigue-resilient biomechanics.

### Risk factors

Multiple prospective and cross-sectional studies have highlighted a multifactorial risk profile for ACL injury in female soccer players, encompassing neuromuscular, biomechanical, anatomical, and strength-related determinants. A large prospective Japanese cohort identified lower hamstrings-to-quadriceps (H/Q) strength ratio, higher quadriceps strength, and longer playing experience as significant predictors of noncontact ACL injury, suggesting a mismatch between quadriceps dominance and insufficient hamstring capacity as a key intrinsic vulnerability in young female athletes.^21^

Similarly, a prospective Spanish cohort evaluating strength and flexibility parameters found that reduced hamstring flexibility, greater quadriceps strength, and deficits in gastrocnemius/soleus flexibility were associated with increased injury risk, reinforcing the role of muscular imbalance and inadequate posterior chain—comprising the hamstrings, gluteal muscles, and lumbar–pelvic extensor musculature—in ACL susceptibility.^22^

Biomechanical assessments consistently demonstrated that high-risk players show altered kinematic patterns. Two- and three-dimensional analyses during cutting tasks revealed that athletes who later sustained ACL injuries exhibited greater knee valgus, increased internal rotation, reduced knee and hip flexion, and contralateral pelvic and trunk drop, all contributing to a high-risk movement strategy during deceleration and directional changes.^16^

Complementary findings from change of direction electromyographic studies showed that players with prior valgus-collapse mechanisms have increased vastus medialis and gastrocnemius activation, reduced tibial rotation control, and altered transverse-plane angular velocity, indicating dysfunctional neuromuscular strategies that may precede ACL injury.^20^

Inter-limb biomechanical asymmetry has also emerged as a relevant risk factor. A motion-capture study of professional players performing drop-vertical jumps identified significant discrepancies in rotational and dorsiflexion ranges of motion, together with asymmetric landing forces, highlighting inadequate load distribution and neuromuscular control as contributors to injury risk in elite female soccer athletes.^19^

This aligns with earlier work showing that poor inter-limb symmetry during functional tasks such as vertical jumping and landing is commonly associated with elevated ACL strain. Anatomical predispositions have also been explored. A five-year prospective registry-based cohort demonstrated that generalized joint hypermobility and knee hyperextension >5° significantly increased the risk for second ACL injuries in female football players with a prior ACL reconstruction, although these factors did not predict primary ACL tears in previously uninjured players.^23^

These findings suggest that laxity-related profiles may primarily influence reinjury vulnerability rather than first-time ACL rupture. Further biomechanical insights come from controlled laboratory studies evaluating kinematics during standardized cutting and turning tasks. High-risk athletes demonstrate greater tibial external-rotation range, slower tibial angular-velocity control, and greater reliance on quadriceps-dominant activation patterns, particularly during cutting tests with larger directional changes, which impose higher stabilization demands on the knee joint.^20^

Similarly, cross-sectional change of direction studies comparing high-risk “valgus-collapse history” players with healthy controls confirmed differences in knee joint ROM, rotational control, and muscle recruitment strategies during change of direction, especially at higher cutting amplitudes.^20^

Additional work reinforces these patterns: investigations in futsal and soccer players have shown that inadequate neuromuscular control, especially during high-velocity changes of direction, is associated with altered hip, knee, and ankle coordination, all of which are recognized contributors to ACL load accumulation.^20^

Moreover, emerging evidence from preseason screening of youth players demonstrated that hip abductor weakness, reduced hip external-rotation strength, and fatigue-related neuromuscular deficits may also contribute to ACL risk, although these findings appear population-specific and are not consistently observed across all cohorts.^21^

Finally, the most recent prospective study on intrinsic Asian female soccer players confirmed that risk profiles differ across populations, with quadriceps dominance and reduced hamstring contribution emerging as stronger predictors in Japanese athletes than in Western cohorts, where joint laxity and hip abductor weakness have traditionally been emphasized.^21^

This underscores the importance of tailoring risk screening strategies to specific geographic cohorts in female football, as biomechanical and epidemiological risk profiles may differ between Asian and Western populations. Collectively, the evidence suggests that ACL injury risk in female soccer players is predominantly driven by quadriceps dominance, weakness or delayed activation of the hamstrings, movement-quality deficits during change of direction and landing, inter-limb asymmetry, and in selected populations, hypermobility-related anatomical profiles. Comprehensive screening and prevention programs should therefore integrate strength balance (particularly Hamstrings-to-Quadriceps strength [H/Q] ratio), neuromuscular movement assessment, and asymmetry evaluation to effectively target this multifactorial risk landscape.

### Role of hormones

Hormonal fluctuations across the menstrual cycle have been proposed as intrinsic contributors to injury susceptibility in female football players, particularly due to the modulatory effects of estrogen and progesterone on ligamentous laxity, neuromuscular control, and musculotendinous tissue properties. Evidence from a large four-year cohort of English international players demonstrated that injury incidence varies across menstrual phases, with the *late follicular phase*—characterized by peak estrogen concentrations—showing a 47% higher overall injury incidence compared with the early follicular phase, and nearly doubled rates of muscle and tendon injuries (incidence rate ratio 1.88).^24^

These findings support prior mechanistic hypotheses that rising estrogen may reduce ligament stiffness and alter tendon mechanical behavior, potentially compromising joint stability during high-load actions. Importantly, the same study identified that 20% of all injuries occurred when players were “overdue” menstruation, suggesting that extended menstrual cycles—a common manifestation of low energy availability or menstrual dysfunction—may represent an additional period of elevated risk. Notably, joint and ligament injuries accounted for a higher proportion of injuries sustained during overdue cycles (36%) compared with those occurring within normal cycle length (21%), indicating a potential interaction between endocrine disruption and tissue vulnerability.^24^

Collectively, available data highlight the menstrual cycle as a meaningful biological modifier of injury risk in elite female football, with the late follicular phase and periods of delayed menstruation emerging as particularly sensitive windows. However, the current evidence base remains limited by reliance on self-reported cycle characteristics, lack of hormonal confirmation, and relatively small numbers of tissue-specific injuries, underscoring the need for future studies integrating precise hormone quantification, verified cycle phase classification, and sport-specific exposure monitoring.

### Imaging

Imaging plays a central role in the evaluation of ACL injuries in adolescent and young female soccer players. In addition to neuromuscular and biomechanical factors, anatomical characteristics have been implicated in ACL injury risk, including a narrower femoral intercondylar notch and increased posterior tibial slope, both of which may increase anterior tibial translation and ACL loading, particularly in female athletes.^25^

Magnetic resonance imaging (MRI) remains the gold standard for diagnosis, allowing detailed assessment of ACL integrity and associated intra-articular damage. A complete ACL tear appears as loss of fiber continuity with high-signal intensity and ligament laxity on fluid-sensitive sequences, frequently accompanied by characteristic bone contusions on the lateral femoral condyle and posterolateral tibial plateau, reflecting the pivot-shift injury mechanism common in female athletes.^25^

MRI also enables evaluation of concomitant injuries, such as meniscal tears or MCL sprains, which are frequent in this population and influence prognosis and management. Early and accurate imaging diagnosis is crucial given the higher susceptibility of postpubertal female soccer players to ACL tears and the elevated risk of reinjury after return to play.

### Graft choice and lateral extra-articular procedures

In female athletes, graft selection should be individualized and based on a balance between mechanical robustness, reinjury risk, and donor-site morbidity. Current evidence suggests lower graft failure rates with bone–patellar tendon–bone (BPTB) autografts compared with hamstring tendon grafts in young women, whereas quadriceps tendon (QT) autografts represent a promising alternative, although high-quality female-specific data remain limited.^26^

Graft selection is a critical consideration in ACLR for female soccer players, who represent a uniquely high-risk subgroup with elevated rates of graft failure and lower return-to-sport (RTS) rates compared with their male counterparts. Multiple cohort studies demonstrate that both BPTB and QT autografts provide reliable functional outcomes in this population. In an all-female soccer cohort with a mean 4.8-year follow-up, Herman et al. reported comparable postoperative International Knee Documentation Committee (IKDC) scores, return-to-soccer rates (78% for BPTB vs 71% for QT), and low revision rates (9% and 0%, respectively), indicating that both grafts are viable options for competitive players.^27^

Hamstring tendon autografts remain widely used in young female soccer players; however, outcome data in this population warrant cautious interpretation. In a cohort of 71 adolescent female soccer players, Britt et al. reported comparable patient-reported outcomes and overall reinjury rates between hamstring and BPTB grafts at mid-term follow-up. Among athletes who returned to soccer, hamstring autografts demonstrated a numerically higher graft failure rate compared with BPTB (22.2% vs 10.3%), although this difference did not reach statistical significance, likely due to limited statistical power.

In addition, hamstring graft recipients achieved significantly lower postoperative Tegner activity scores, suggesting a potential impact on return to high-demand sporting activity. Fear of reinjury was also more frequently reported in the hamstring cohort as a reason for not returning to preinjury level of play.

Although current female-specific datasets do not allow definitive conclusions regarding graft superiority, these findings contribute to ongoing concerns regarding hamstring autografts in high-risk female soccer players and support careful graft selection based on individual risk profile, sporting demands, and concomitant stabilizing strategies.^28^

Fear of reinjury emerged as a dominant factor in failure to RTS in both graft groups, highlighting the relevance of psychological readiness when evaluating graft success. Beyond graft selection, growing evidence underscores the importance of LEAPs, particularly in high-risk female athletes. Editorial commentary by van der List highlights that female soccer players historically demonstrated failure rates approaching 27% with isolated ACLR and that the reemergence of augmenting procedures such as lateral extra-articular tenodesis (LET) or anterolateral ligament reconstruction has contributed to improved rotational stability and reduced graft failures over the past decade.^29^ This aligns with contemporary data showing that high-risk features—such as generalized ligamentous laxity, increased pivot-shift grade, and valgus–internal rotation loading patterns—call for strategies beyond isolated soft-tissue grafts.

The largest female-specific dataset provided evaluating LET comes from Parmar et al., who examined 133 competitive female soccer players undergoing primary ACLR with or without LET. Despite the LET group exhibiting markedly greater generalized ligamentous laxity (48.8% vs 18.9%) and higher preoperative pivot-shift grades, graft failure rates (4.7% vs 3.0%) and RTS rates (90.7% vs 85.6%) were similar between cohorts, suggesting that LET effectively mitigates the additional risk posed by ligamentous laxity.^30^

LET augmentation also resulted in a significantly higher proportion of patients achieving a grade-0 postoperative pivot shift, supporting its role in restoring rotational stability. Taken together, these findings indicate that BPTB and QT autografts both provide acceptable mid-term outcomes in female soccer players, whereas hamstring autografts appear less favored due to concerns about higher failure rates in this demographic, consistent with prior large-scale literature. In athletes with generalized ligamentous laxity, high pivot-shift grades, or other biomechanical risk factors, the incorporation of LET or other LEAP appears to normalize outcomes, reducing the historically elevated failure rates observed in this subgroup. Contemporary expert opinion further emphasizes avoiding isolated soft-tissue grafts in high-risk female players and adopting LEAP when risk profiles or intraoperative findings warrant augmentation.^29^

### Return to play in female soccer players

Return to play after ACLR in female soccer players shows substantial variability across competition levels, with generally high rates of return but often reduced performance and persistent functional or psychological limitations. In the National Women's Soccer League, Abed et al. reported a 90% RTP rate, with a median time of 12.1 months, although players demonstrated a significant decrease in percentage of minutes played and reductions in goals and assists during the first year after RTP.^2^

At the elite European level, Ekstrand et al. documented that ACL injuries represent one of the most time-consuming injuries, with severe knee injuries—including ACL tears—accounting for some of the longest absence durations in UEFA Women's Elite Club Football; ACL injuries specifically were among the top four diagnoses responsible for 35% of all time lost.^31^

Consistently, Bloch et al. showed that female footballers in German professional leagues not only have higher ACL injury prevalence but also experience longer time loss and delayed surgical care compared with men, highlighting important sex-specific disparities in clinical management and time to RTP.^32^

Performance-based assessments reveal that even when female players return to the field, movement strategies and biomechanics differ from uninjured peers. Kowalczyk et al. found that collegiate female soccer athletes post-ACLR achieved comparable jump height and reactive strength index to controls but relied on hip-dominant compensatory strategies and demonstrated persistent deficits in knee power generation, indicating that RTP does not necessarily equate to restored biomechanical function.^33^

Similarly, Fältström et al. reported that female players who sustained either primary or secondary ACL injuries showed reduced self-reported knee function, lower activity levels, and decreased satisfaction over a five-year follow-up period, suggesting a long-term functional burden even after successful RTP.^34^

Prospective data further show that specific physical performance measures do not reliably predict safe RTP. In a two-year cohort, Fältström et al. demonstrated that players who went on to sustain either a primary or secondary ACL injury actually exhibited better hop performance—longer jumps and more repetitions—than injury-free players, challenging traditional assumptions that hop test symmetry alone is a sufficient benchmark for RTP readiness.^35^

Broader epidemiological evidence reinforces that return to previous levels of football may be influenced by psychological readiness and confidence; male players exhibit higher IKDC, Knee injury and Osteoarthritis Outcome Score (KOOS), ACL–Quality of Life (ACL-QoL) questionnaire, and ACL–RTS after Injury scale (ACL-RSI) scores than females, and female athletes often report more apprehension when returning to play.^36^

Finally, the most comprehensive synthesis to date confirms that approximately 69% of female athletes RTS after ACLR at an average of 10.8 months, although the quality of available evidence remains limited and reasons for failing to RTP—often related to fear of reinjury—are underreported in the literature.^37^

### Psychological aspects

Psychological factors represent a critical yet often underappreciated component in the risk, recovery, and RTS trajectory of female soccer players following ACL injury. Evidence consistently indicates that personality traits, stress responses, and cognitive–motor interactions can influence both injury mechanisms and postinjury outcomes.

A large cross-sectional study evaluating personality traits and perfectionism in soccer players with and without ACL reconstruction found no meaningful differences in personality profiles among females, suggesting that personality-based psychological predispositions may *not* explain ACL injury occurrence in women.^38^

This contrasts with male players, in whom small differences were observed, but with unclear clinical significance. However, prior literature referenced within the same study reports that traits such as somatic trait anxiety, stress susceptibility, mistrust, and irritability have been associated with general injury risk in soccer players, highlighting that psychological stress responses remain relevant even if not sex-specific.

Beyond personality, cognitive load and decision-making pressures appear highly influential in ACL injury mechanisms in female soccer players. Complex, reactive, match-like situations requiring divided attention and rapid inhibition of preplanned actions may impair biomechanical control. A biomechanical study demonstrated that increasing cognitive demands—by expanding movement options and using an opponent-based reactive change of direction task—significantly altered proximal segment kinematics, including pelvis tilt/rotation and trunk rotation, even though knee joint mechanics were not directly affected.^18^

These findings align with prior video-analysis evidence indicating that ACL injuries frequently occur during cognitively demanding situations such as pressing, tackling, or reacting to feints, where attentional shifts may compromise neuromuscular coordination.

Psychological readiness after ACL reconstruction is another domain of high relevance. Fear of reinjury, low confidence, and emotional dysregulation have been well documented as barriers to successful RTS outcomes after ACL-injury. Indirectly, measures such as psychic anxiety—which was found to be higher in female players compared to males—may represent tendencies toward increased emotional reactivity under competitive stress, potentially influencing rehabilitation experiences and decision-making during return-to-play progression.^38^

Additionally, perfectionistic tendencies, including personal standards, concern over mistakes, and perceived coach/parental pressure, showed small sex-based differences and may influence coping strategies and self-evaluation during recovery. High perfectionistic strivings have been previously associated with both enhanced engagement and increased vulnerability to stress; thus, their role in postinjury behavior in female players warrants further exploration.

Finally, psychological contributors also interact with cognitive–motor interference, particularly during high-speed, unanticipated movements. Under elevated cognitive load, athletes may experience compromised motor execution and suboptimal trunk and pelvic control, indirectly heightening injury susceptibility—even in the absence of knee-specific deficits—highlighting the need for neurocognitive-based training interventions in ACL prevention programs for female soccer players.^18^

### Professionalization mismatch

Recent evidence highlights the concept of a *professionalization mismatch* in women's football, referring to the widening gap between the rapidly increasing physical, competitive, and commercial demands placed on female players and the insufficient structural, medical, and organizational support available to them. Despite accelerated professionalization over the past decade—with more women holding full-time contracts and earning salaries entirely from football—the infrastructure surrounding women's teams often remains inadequate. The viewpoint by Le et al. (2025) reports that many players still compete under precarious working conditions, including short-term contracts (often 12–18 months), financial instability, dense match calendars, extensive travel demands, and reduced recovery windows.^39^ This environment increases cumulative physical stress while limiting access to multidisciplinary medical teams and high-quality rehabilitation resources.

Survey data presented in the same viewpoint underscore this structural deficit: for example, players in the Women's National League reported the absence of a fully qualified physiotherapist on staff, and up to one-third of players in the 2023 World Cup qualifying tournaments experienced inadequate or nonexistent recovery facilities. Similar concerns arise regarding limited medical care, insufficient training load monitoring, and logistical constraints such as staffing shortages and restricted facility access, all of which directly impact players’ physical readiness and injury resilience.^39^

This misalignment between rising performance expectations and limited support likely contributes to the persistently high incidence of ACL injuries in women's football. Although professionalization should theoretically lead to improved conditioning, better resource allocation, and enhanced medical supervision, the current shortfall in structural support may instead increase players’ exposure to high-risk situations without adequate preventive frameworks. As highlighted by Le et al., professional women's football still lacks rigorous, context-specific ACL injury reduction strategies, and very few randomized or high-quality longitudinal trials have evaluated the effectiveness of prevention programs in elite female players. Existing programs such as Fédération Internationale de Football Association (FIFA) 11+ are widely adopted at the amateur level, but their suitability for professional athletes—who face higher intensities, condensed schedules, and unique psychosocial pressures—remains uncertain.^39^ Overall, the professionalization mismatch represents a critical systemic risk factor for ACL injury in female soccer players, emphasizing the need for tailored injury-prevention strategies that account for workload demands, resource limitations, and the specific realities of elite women's football.

### Long-term outcomes

Long-term outcomes after ACL injury in female soccer players are characterized by a very high burden of radiographic OA, persistent symptoms, and functional limitations. The landmark cohort study by Lohmander et al. followed 103 women who sustained an ACL tear while playing soccer and evaluated them 12 years after injury. Among the 67 participants who underwent standardized weight-bearing radiographs, 82% showed radiographic changes suggestive of early or established OA, and 51% fulfilled criteria for definite radiographic knee OA, approximating Kellgren–Lawrence grade ≥2.^38^ Importantly, radiographic OA was predominantly located in the tibiofemoral compartment, although 13% of players also demonstrated patellofemoral involvement. Meniscal injury played a significant role: individuals who had undergone meniscal surgery at the time of ACL injury had a markedly higher prevalence of radiographic OA (69%) compared with those without meniscal surgery (39%).^40^

Symptomatically, the long-term burden was substantial. Based on KOOS thresholds for clinically relevant symptoms, 75% of the cohort was symptomatic, and 42% met criteria for symptomatic radiographic OA. KOOS subscale scores showed major impairments particularly in *sports and recreation* and *knee-related QoL*, both with mean values of 54/100, compared with 89/100 in age-matched uninjured reference players.^40^

Half of the athletes reported having significantly modified their lifestyle, and 70% lacked confidence in the previously injured knee. Regarding the influence of treatment, ACL reconstruction did not reduce the long-term risk of radiographic OA nor the likelihood of being symptomatic. Women who underwent surgical reconstruction had OA rates similar to those treated nonoperatively (56% vs 42%, *p* = .3), and multivariable adjusted analyses confirmed no protective effect of reconstruction. Reconstruction was, however, associated with a higher prevalence of patellofemoral degenerative changes (61% vs 28%).^40^

Functionally, only 8% of players were still participating in organized soccer 12 years after injury, and fewer than 15% had activity levels comparable to preinjury. This illustrates the profound long-term impact of ACL injury on sport participation and daily function in female soccer players.

Overall, Lohmander et al. highlight that ACL injury in young female athletes places them at exceptionally high risk of early-onset knee OA, persistent symptoms, and reduced QoL, underscoring the urgent need for improved prevention, early management, and joint-preserving strategies in this population.^40^

### Prevention

A growing body of evidence highlights that ACL injury prevention in female soccer players requires integrated strategies addressing neuromuscular, biomechanical, cognitive, and sex-specific risk factors. Despite robust evidence supporting prevention programs, real-world implementation and adherence remain insufficient, limiting their overall effectiveness on injury reduction.

Exercise-based ACL injury prevention programs—particularly FIFA 11+, FIFA 11+ Kids, PEP, and similar neuromuscular training protocols—have consistently demonstrated positive effects on modifiable biomechanical risk factors. In preadolescent female players, an eight-week FIFA 11+ Kids intervention improved knee flexion and hip internal rotation control during single-leg drop landing, thereby reducing kinematic patterns associated with ACL loading.^41^

Similarly, unilateral strength-focused interventions have been shown to enhance change of direction mechanics—one of the most common injury mechanisms in female soccer—reducing knee valgus, trunk instability, and poor foot placement patterns. After a 10-week individualized unilateral strength program, high-risk athletes decreased from 82% to 0%, highlighting the value of individualized neuromuscular conditioning.^42^

Fatigue and unpredictable game situations are important determinants of injury risk. Exposure to unanticipated pivoting and turning significantly alters lower-limb biomechanics, increasing hip and knee joint loads and elevating ACL stress; fatigue amplifies these effects by impairing neuromuscular control during high-demand movements.^17^

These findings support the incorporation of the integration of cognitive–motor training (i.e. motor exercises combined with concurrent cognitive tasks such as decision-making, reaction time, or visual–spatial processing) into injury-prevention programs.

At the youth level, improving implementation is a major challenge. A prospective cohort study in amateur girls’ soccer demonstrated that a knowledge-to-action strategy for coach education produced significantly higher implementation rates of ACL injury prevention programs compared to passive educational handouts.^43^

Notably, performing an ACL prevention program at least twice per week reduced ACL injury risk by 85%, demonstrating the importance of consistency. However, large-scale European survey data reveal that although awareness of ACL injuries is high among key stakeholders—namely players, coaches, and medical and performance staff—only 22% of players and 51% of coaches report using structured injury-prevention programs, particularly at the grassroots level (i.e. amateur, youth, and nonelite football settings), highlighting a substantial implementation gap and reinforcing the need for targeted educational interventions.^44^

Newer prevention models emphasize precise identification of intrinsic risk factors. A randomized clinical trial protocol proposes a targeted neuromuscular intervention specifically for female players with dynamic knee valgus, integrating biomechanical screening and tailored exercises.^45^

This personalized approach may address limitations of universal prevention programs, especially for players with persistent high-risk movement patterns.

Broader considerations also influence injury prevention success. A narrative review highlighted that concussion-related neuromuscular deficits—such as altered proprioception, delayed reaction time, and compromised postural control—may increase ACL injury susceptibility and should be integrated into prevention and RTS decision-making.^46^

A public health perspective emphasized the financial, performance, and long-term joint-health impact of ACL injuries in female soccer players, reiterating the importance of developing effective prevention infrastructures at club and federation levels.^47^

Finally, a narrative review underscored the need for sex-specific injury prevention strategies, as existing programs show lower effectiveness in women than in men, partly due to hormonal influences, strength imbalances, and neuromuscular differences; tailored programs may better address these biological and biomechanical characteristics.^48^

Overall, current evidence suggests that ACL injury prevention in female soccer players is most effective when programs are:

1. *Neuromuscular-focused*, addressing strength, biomechanics, agility, and landing mechanics;
2. *Individualized*, particularly for athletes with dynamic valgus, strength asymmetries, or poor neuromuscular control;
3. *Implemented consistently*, at least twice weekly;
4. *Supported by structured coach education*, improving adherence and transfer into practice;
5. *Expanded to include cognitive–motor and fatigue-based scenarios*, mirroring real-game demands;
6. *Integrated with sex-specific considerations*, including hormonal influences and concussion-related neuromuscular deficits.

Such strategies collectively represent the most comprehensive and evidence-based approach to reducing ACL injuries in female soccer players.

## Clinical implications

### For orthopedic surgeons

- Consider anatomical risk modifiers (e.g. notch width, tibial slope) during preoperative planning.
- For hyperlax athletes, combine ACL reconstruction with LEAP to enhance rotational control
- In female players with small patellae or a predisposition to anterior knee pain, QT autografts may represent a preferable option, whereas the use of hamstring tendon autografts should be individualized in light of potential effects on posterior chain strength.
- Incorporate *psychological readiness assessments* before clearance to play.

### For rehabilitation specialists

- Focus on movement retraining emphasizing trunk, hip, and knee control rather than simple strength symmetry.
- Use cognitive dual-task drills and reactive agility to simulate match conditions.
- Monitor H/Q ratio and eccentric hamstring strength, as these are modifiable and predictive parameters
- Adopt validated RTS criteria integrating functional tests, self-report measures, and biomechanical screening.

### For coaches and federations

- Promote the regular use of preventive warm-up programs (e.g. FIFA 11+) at least twice weekly.
- Implement periodized load management considering menstrual cycle, fatigue, and fixture congestion.
- Promote education and surveillance systems to ensure compliance and accountability.
- Encourage multidisciplinary collaboration among medical staff, coaches, and strength specialists.

## Research gaps

Despite significant progress, several key gaps remain:

1. Underrepresentation of female cohorts—Women constitute <20% of participants in ACL research, limiting sex-specific inference.
2. Lack of standardized outcome measures—RTS definitions vary, hindering cross-study comparison.
3. Insufficient hormonal profiling—Menstrual phase and contraceptive use are often unreported.
4. Sparse long-term data—Few studies extend beyond 5–10 years, leaving OA risk uncertain.
5. Limited access to elite populations—Most biomechanical data derive from subelite or collegiate athletes.
6. Psychological integration—RTS and prevention research rarely include validated psychological instruments.
7. Implementation science—Barriers to adherence remain underexplored compared to program efficacy.

Addressing these gaps requires collaborative, multicenter designs and equity-driven funding to prioritize female athlete health.

## Future directions

The next frontier in ACL injury research lies in individualized, technology-assisted prevention and rehabilitation. Artificial intelligence and wearable motion sensors will soon allow real-time detection of maladaptive biomechanics, enabling proactive correction. Virtual reality and augmented feedback systems can simulate complex, decision-heavy environments that mirror real soccer demands.

In the clinical realm, integrated care models—combining orthopedic, physiotherapeutic, psychological, and nutritional expertise—should become standard for elite female athletes. Moreover, the sport must embrace *gender-specific training paradigms* that consider hormonal fluctuations, pelvic morphology, and sociocultural context.

Finally, the narrative surrounding ACL injury in women's soccer must shift from inevitability to preventability. With adequate research investment, policy enforcement, and interdisciplinary cooperation, the disproportionate ACL burden in female soccer can—and should—be reduced within the next decade.

## Conclusions

ACL injury in female soccer players remains a major and multifactorial challenge in sports medicine. The available evidence indicates that biomechanical, neuromuscular, hormonal, psychological, and contextual factors interact to create a uniquely high-risk profile in this population.

Although surgical reconstruction enables RTS in most athletes, persistent functional deficits, fear of reinjury, and elevated rates of secondary injury highlight the need for more individualized and sex-specific management strategies. Prevention programs are effective when consistently implemented, yet their real-world impact is limited by adherence, contextual constraints, and insufficient tailoring to elite female players.

Future progress will depend on integrated approaches combining high-quality research, individualized rehabilitation, psychological support, and structural investment in women's football. With coordinated, evidence-based efforts, the burden of ACL injury in female soccer players can be meaningfully reduced.

## Key messages

- ACL injury risk in female soccer players emerges after puberty and is influenced by region-specific biomechanical and anatomical profiles, underscoring the need for tailored screening strategies.
- Psychological factors, including fear of reinjury and psychological readiness, play a central role in RTS outcomes and should be systematically integrated into rehabilitation pathways.
- In female athletes, graft selection should be individualized; BPTB autografts show lower failure rates in young women, QT represents a promising alternative, and LET may enhance stability in hyperlax players.
- Hamstring tendon autografts warrant careful consideration in female players due to the potential impact on posterior chain function and neuromuscular control.
- Despite strong evidence supporting neuromuscular injury-prevention programs, real-world adoption remains limited, particularly in amateur, youth, and nonelite football settings.
- The gap between awareness and implementation represents a major, modifiable determinant of ACL injury burden in women's soccer.
- Future prevention strategies should move beyond generic programs and incorporate cognitive–motor training, psychological readiness assessment, and technology-assisted monitoring to better reflect the demands of the modern game.

## Supplemental Material

sj-docx-1-sci-10.1177_00368504261428994 - Supplemental material for ACL injury in female soccer players: Risk, resilience, and prevention in the modern game Supplemental material, sj-docx-1-sci-10.1177_00368504261428994 for ACL injury in female soccer players: Risk, resilience, and prevention in the modern game by Riccardo D’Ambrosi, Philipp Baumert and Christian Fink in Science Progress
