# Supplementary material for: ACL injury in female soccer players: Risk, resilience, and prevention in the modern game
Source: Sci Prog. 2026 Apr 9;109(2):00368504261428994. doi: 10.1177/00368504261428994 (PMC13070141; doi:10.1177/00368504261428994)
Supplement: sj-docx-1-sci-10.1177_00368504261428994 - Supplemental material for ACL injury in female soccer players: Risk, resilience, and prevention in the modern game [file sj-docx-1-sci-10.1177_00368504261428994.docx]

**Supplementary Table 1. Database-specific overview of literature search and study selection.**
This table summarizes the number of records identified from each database, duplicates removed, records screened at title and abstract level, full-text articles assessed, and articles excluded after full-text review, together with the main reasons for exclusion. The overview is provided to enhance transparency of the literature search and selection process within the narrative design of the review.

| **Database** | **Records identified** | **Duplicates removed** | **Records screened (title/abstract)** | **Full-text articles assessed** | **Articles excluded after full-text** | **Main reasons for exclusion** |
| --- | --- | --- | --- | --- | --- | --- |
| PubMed/MEDLINE | 64 | 14 | 50 | 41 | 9 | Non–soccer cohorts; mixed-sex data without female stratification; non-ACL outcomes |
| Scopus | 7 | 3 | 4 | 3 | 1 | Editorials; conference abstracts; biomechanical studies without clinical relevance |
| Web of Science | 5 | 2 | 3 | 2 | 1 | Narrative papers with overlapping content; insufficient sport-specific data |
| **Total** | 76 | 19 | 57 | 46 | 11 | — |
